# Video-Based Pulse Rate Variability Measurement Using Periodic Variance Maximization and Adaptive Two-Window Peak Detection

**DOI:** 10.3390/s20102752

**Published:** 2020-05-12

**Authors:** Peixi Li, Yannick Benezeth, Richard Macwan, Keisuke Nakamura, Randy Gomez, Chao Li, Fan Yang

**Affiliations:** 1 ImViA-EA7535, Univ. Bourgogne Franche-Comté, 21000 Dijon, France; yannick.benezeth@u-bourgogne.fr (Y.B.); fanyang@u-bourgogne.fr (F.Y.); 2 LPL, CNRS-UMR7538, Univ. Paris 13, 93430 Villetaneuse, France; Richard.macwan@univ-paris13.fr; 3 Honda Research Institute Japan Co., Ltd., 8-1 Honcho, Wako-shi, Saitama 351-0114, Japan; keisuke@jp.honda-ri.com (K.N.); r.gomez@jp.honda-ri.com (R.G.); 4 State Key Laboratory of Acoustics, Institute of Acoustics, Chinese Academy of Sciences, Beijing 100190, China; lichao8601@hotmail.com

**Keywords:** remote photoplethysmography (rPPG), video analysis, pulse rate variability (PRV), periodic variance maximization (PVM), peak detection

## Abstract

Many previous studies have shown that the remote photoplethysmography (rPPG) can measure the Heart Rate (HR) signal with very high accuracy. The remote measurement of the Pulse Rate Variability (PRV) signal is also possible, but this is much more complicated because it is then necessary to detect the peaks on the temporal rPPG signal, which is usually quite noisy and has a lower temporal resolution than PPG signals obtained by contact equipment. Since the PRV signal is vital for various applications such as remote recognition of stress and emotion, the improvement of PRV measurement by rPPG is a critical task. Contact based PRV measurement has already been investigated, but the research on remotely measured PRV is very limited. In this paper, we propose to use the Periodic Variance Maximization (PVM) method to extract the rPPG signal and event-related Two-Window algorithm to improve the peak detection for PRV measurement. We have made several contributions. Firstly, we show that the newly proposed PVM method and Two-Window algorithm can be used for PRV measurement in the non-contact scenario. Secondly, we propose a method to adaptively determine the parameters of the Two-Window method. Thirdly, we compare the algorithm with other attempts for improving the non-contact PRV measurement such as the Slope Sum Function (SSF) method and the Local Maximum method. We calculated several features and compared the accuracy based on the ground truth provided by contact equipment. Our experiments showed that this algorithm performed the best of all the algorithms.

## 1. Introduction

The contact photoplethysmography (PPG) devices used by hospitals and research labs are small photoelectric sensors that measure the blood volume pulse (BVP) through fingers or ears by detecting the change of light intensity which passes through the tissue. This technique is widely used in medical applications for its benefits of being low cost and high convenience compared with an electrocardiogram (ECG). The remote photoplethysmography (rPPG) has recently attracted significant attention because it is a non-contact method to measure physiological parameters and can be used in long-term and non-interruptive monitoring. The basic principle behind rPPG derives from that of contact PPG where instead of using a photoelectric sensor, rPPG uses an RGB camera. The variation in the light intensity reflected by the human face can be used to measure BVP, which in turn can be used to estimate physiological parameters such as Heart Rate (HR) and Pulse Rate Variability (PRV). Heart Rate Variability (HRV) is defined as the variation of the inter-beat intervals measured by the distance of the R-peaks in the ECG signal [1]. HRV has been studied intensively by biomedical researchers as a sign of cardiac health [2] and Autonomic Nervous System (ANS) [3] which can be used to reflect human stress and emotion [4]. According to the previous research, the Pulse Rate Variability (PRV) measured by PPG techniques can be a surrogate measurement of HRV in some conditions [5]. And Poh et al. showed that high degrees of agreement were achieved between the PRV measured by a contact sensor and a Webcam [6]. This means that PRV measured by rPPG has great potential for multimedia applications such as remote assessment of pain, stress and emotion, although it cannot replace HRV in critical medical analysis due to the low frame rate, the noise and errors of rPPG method and completely different experimental conditions.

According to existing studies, the accuracy of remote HR measurement can be higher than 90% [7]. However, PRV measurement with rPPG is more complicated, because it requires precise peak detection of the BVP signal, which is prone to noise emanating from sensors, light illumination variation, movements, and so forth. Although some methods exist to improve the BVP peak detection and PRV measurement for contact PPG [8,9,10], most of them have not been applied in rPPG applications. Some works used complex equipment such as thermal cameras and 5-band cameras to improve the performance of remote PRV measurement [11,12], but such equipment is either too expensive or not widely available in everyday life. Another issue is that the existing remote PRV research works usually assess the accuracy of measured PRV through stress detection or emotion recognition instead of evaluating the performance with respect to the ground truth PRV signal obtained from a contact sensor. For instance, Macduff et al. [13] and Mitsuhashi et al. [14] used rPPG methods for stress/emotion recognition where the results showed an accuracy of 60%–85% for emotion prediction via remote PRV. But the errors between the remotely measured PRV and the ground truth were not studied and discussed. None of the work which adopted the low-cost RGB camera has been able to recognize the emotion states with the accuracy higher than 90%. Therefore, improving the remote PRV measurement and assessing it with quantitative ground truth is an important task.

In this paper, we use the dataset generated by a low-cost 3-band web camera and adopted the newly proposed Periodic Variance Maximization (PVM) method [15] to extract the BVP signal. Then, we used an event-related Two-Window [16] algorithm for BVP peak detection to increase the accuracy of PRV measurement via rPPG. To dynamically adapt to the observed data, we propose a method to adaptively set the parameters for the Two-Window peak detection. We compare our algorithm with other peak detection methods using several assessment metrics and show that this method performs the best of all. Section 2 describes related work, followed by proposed method in Section 3. Section 4 and Section 5 describe the experiments and results, followed by the conclusion in Section 6.

## 2. Related Works

In this section, the related works are described in three research directions: (1) Stress and emotion detection with rPPG framework; (2) Remote PRV measurement improvement with novel cameras; (3) Improvement of BVP peak detection and HRV/PRV measurement with contact equipment.

### 2.1. Stress and Emotion Detection with rPPG Framework

As explained, the rPPG can be used for remote detection of stress and emotion. Macduff et al. [13] proposed a framework that adopted facial landmarks to get the region of interest (ROI) from the face, and used ICA to process and combine the RGB signals to get the BVP signal, and then obtained PRV based on the extracted BVP signal. They then utilized several frequency features of PRV such as High Frequency (HF) power, Low Frequency (LF) power and LF/HF to train the model on the dataset which included two emotion states, relaxed and stressed states. It turned out that PRV alone could achieve an accuracy of 70% to distinguish between these two states. Mitsuhashi et al. [14] proposed a method that combined the two-layer (melanin and hemoglobin layers) model and singular valued decomposition in RGB space for pulse signal measurement from videos. They defined three different stress levels by the difficulty of the tasks. For instance, easy (mental arithmetic of 5*6), middle (mental arithmetic of 13*16), and difficult (mental arithmetic of 114*123). They used the KNN method to classify the stress modes. The results showed that the accuracy of the classification is around 66%–83% for different stress levels. Belaiche et al. [17] used both micro-expressions and PRV to predict three emotion states, namely, happiness, disgust and anger. This study found that although the accuracy of the PRV based method is higher than the micro-expressions based method in emotion states recognition but the average accuracy is usually not higher than 60% for the dataset that contains sudden emotion change.

### 2.2. Improving the Remote PRV Measurement with Novel Cameras

An approach to get more precise PRV measurement is to make use of novel experimental equipment and materials to achieve better performance. Gupta et al. [18] proposed a system that used a thermal camera, a monochrome camera with a color filter and an RGB camera to extract the BVP signal. This novel system was proved quite effective to reduce the noise caused by motion and light illumination variation. With this system, one can monitor the HR and PRV and visualize the data in a real-time scenario. The RGB cameras are the most widely used cameras available in everyday life, however, some researchers suggested that the 5-band RGBCO cameras could work better since more information could be used in the ICA and PCA methods to combine the signals. Mcduff et al. [11] presented a work that adopted the novel 5-band camera and found that the cyan, green, and orange (CGO) bands performed better than RGB bands in measuring the PRV in the frequency domain. The correlation between the signals measured by the contact sensor and the camera was over 90%. Their further work [12] utilized the CGO bands and conducted an experiment which included two randomized-order tasks. The results demonstrated that the PRV features extracted by CGO bands could distinguish between the relaxed and stressed modes with an accuracy of 70%–80%. Additionally, they showed that the value of PRV features could capture the changes of stress for individuals since the two tasks in the experiments caused different stress levels for the participants and they were detected with correlated value change. In the realistic applications, the remote PRV measurement may suffer from missing observations caused by subject movement and subject getting obscured by an object, they addressed the issue by proposing an algorithm to fuse partial camera signals generated from an array of cameras and they improved the PRV measurement in the scenarios where significant amount of data is lost [19]. Fukunishi et al. [20] proposed to use a new ROI detection, a new rule-based method and a new filtering algorithm to improve the performance of the 5-band camera in PRV measurement and they successfully reduced the noise of PRV features in the frequency domain.

### 2.3. Improvement of BVP Peak Detection and HRV/PRV Measurement with Contact Equipment

The remote measurement of PRV can be improved with novel algorithms for BVP peak detection. This improvement has been the focus of biomedical research, especially on signals generated by Electrocardiography (ECG) and contact PPG sensor. Béres et al. [21] gave a comprehensive study of the adequate sampling frequency for contact PPG measurement and the detailed instructions for interpolation and sampling. Zong et al. [22] proposed to use Slope Sum Function (SSF) to enhance the rising part of the ECG signal and reduce the falling part so that the shape of the signal is simplified and more clear to conduct peak detection. Jang et al. [9] adopted this method and used it in the contact PPG. It turned out the SSF could work well on contact PPG signal with some pre-processing and post-processing methods. Computer vision researchers Li et al. [23] further showed that this algorithm could improve the performance of remote PRV measurement despite completely different experimental conditions in the non-contact scenario. The contact PPG measurement under tropical conditions is difficult especially after exercise. Elgendi et al. [16] used an event-related method to solve this problem. This algorithm utilized the property of the PPG signal that the average height of the systolic peak period should be higher than the beat period and the systolic peaks are the highest points inside the peak periods. Thus the method detected the BVP peaks and addressed the non-stationary effects caused by severe exercise conditions in hot and humid environment. The results showed that this method detected the peaks with the sensitivity of nearly 100%. However, the parameters of this algorithm have to be optimized by a brute force search, so it is very time-consuming.

### 2.4. Summary of the Related Works

According to these related works, it can be concluded that: (1) the basic framework of rPPG measurement has been intensively studied and widely adopted. (2) Some computer vision researchers used more complex cameras to improve the rPPG performance, however, the equipment is either expensive or not widely available in everyday life. (3) The majority of the rPPG works do not focus on improving BVP peak detection, which is critical for PRV measurement. (4) Biomedical and signal processing researchers used some novel algorithms to improve the contact PPG measurement, and some of the algorithms have not been adopted by computer vision researchers, possibly due to completely different experimental conditions. (5) Some existing algorithms [16] for peak detection are time-consuming.

In this paper, we adopt a dataset that was recorded by a widely available low-cost camera and utilized the algorithms that could be specifically used in the BVP extraction and peak detection in remote conditions and we show that our framework indeed improved the precision of remotely measured PRV.

## 3. Method

### 3.1. RPPG Signal Extraction with PVM Method

Periodic Variance Maximization (PVM) with Generalized Eigenvalue Decomposition (GEVD) [15] was adopted to process RGB signals and obtain the BVP signal. This algorithm combines PCA with periodicity maximization to extract the quasi-periodic component with unknown period. PVM uses generalized eigenvalue decomposition to obtain a periodicity maximizing basis at a given frequency. This process is then iterated over the human heart rate range to obtain the frequency exhibiting the highest global periodicity. The GEVD step is applied on the pair of covariance and lagged covariance matrices which encapsulate the idea of periodicity. Intuitively, a periodic signal shall exhibit high similarity to its lagged version, if this lag is close to its effective period. This intuition is quantified using a periodicity metric, ρ, described below. Let **x**(i) be the temporal RGB signal at time *i* after centering and detrending. The covariance matrices and lagged covariance matrices are defined as:

(1) $$C_{x}=\frac{1}{N}\sum_{i=1}^{N}x\left(i\right)x{\left(i\right)}^{T},P_{x}=\frac{1}{N}\sum_{i=1}^{N}x\left(i\right)x{\left(i+\tau \right)}^{T},$$

respectively, where x(i+τ) is the signal that lagged by τ seconds. Then GEVD is applied on $P_{x}$ and $C_{x}$ and the generalized eigenvector corresponding to the highest generalized eigenvalue w is used to obtain the signal with highest periodic variance at a given lag τ using:

(2) $$y\left(i\right)=w^{T}x\left(i\right).$$

In practice, the GEVD is applied on symmetrized versions of $P_{x}$ and $C_{x}$ to ensure positive generalized eigenvalues, using $(P_{x}+P_{x}^{T})/2$, which effectively represents the two-way variance among the RGB channels. GEVD aims to estimate the matrix of generalized eigenvectors $W=[w_{1},w_{2},w_{3}]$ and the generalized eigenvalues $D=diag(\lambda_{1},\lambda_{2},\lambda_{3})$ that satisfy:

(3) $$W^{T}P_{x}W=D,W^{T}C_{x}W=I.$$

Denoting w as the generalized eigenvector corresponding to the highest generalized eigenvalue in **D**, the extent of periodic information in y(i) can be quantified as:

(4) $$\rho \left(\tau ,w\right)=\frac{\sum_{i=1}^{N}y\left(i\right)y\left(i+\tau \right)}{\sum_{i=1}^{N}y{\left(i\right)}^{2}}=\frac{w^{T}P_{x}w}{w^{T}C_{x}w},$$

where ρ is the periodicity metric. If the component signals were fully periodic, ρ would be 1 given that $C_{x}$ and $P_{x}$ were equal. This periodicity metric is then optimized over the human heart rate range to obtain $\tau^{*}$, the period corresponding to the BVP, where at each τ the optimum weighting matrix w is obtained by GEVD.

We have extensively evaluated this algorithm and compared it with other methods such as Green, PCA, ICA and Chrominace and the results showed that this algorithm performed the best for extracting the rPPG signal [15].

### 3.2. Adaptive Two-Window Peak Detection

The BVP signal is considered to have two important time windows. The first one is the “beat period”, which is the entire period of one heart beat. And the other is the "systolic peak period", which is the period where a systolic peak appears. A systolic peak period is inside a beat period, so the time length of the systolic peak period is smaller than the beat period. The two periods have two important physical properties: firstly, the average signal amplitude of the systolic peak period is usually higher than the average amplitude of the beat period, and secondly, the systolic peaks are supposed to be the highest points within the systolic peak periods. With the definitions of the two periods and the properties, an event-related algorithm was proposed by Elendi et al. [16] to reduce the noise of systolic peak detection with contact PPG in tropical conditions after exercise. We consider this method useful to reduce the noise caused by the light illumination variation, movement, sensors, and so forth, in the rPPG method.

However, there are two disadvantages of the original event-related method: (1) It adopted too many parameters. (2) Brute force search was used to set all the parameters, namely the frequency band, the duration of the beat and peak periods, and the offset between the peak area’s amplitude and beat area’s amplitude. Therefore the method is very time-consuming and not practical in realistic applications. To address this issue, we propose a fast version of the event-related algorithm that uses two adaptively determined parameters and this algorithm was named as “Adaptive Two-Window Peak Detection”:

Let $W_{b}$ be the window size of the “beat period”, and the moving average of beat period is defined as:

(5) $$MA_{b}\left(i\right)=\frac{1}{W_{b}}(y\left(i-W_{b}/2\right)+\ldots +y\left(i\right)+\ldots +y\left(i+W_{b}/2\right)),$$

where y(i) is the BVP value at time *i*. $W_{b}$ can be the time length of the window, or the number of time stamps of the window if the signal is discrete. Similarly, let the $W_{p}$ be the window size of “systolic peak period”, and the moving average of the systolic peak period is defined as:

(6) $$MA_{p}\left(i\right)=\frac{1}{W_{p}}(y\left(i-W_{p}/2\right)+\ldots +y\left(i\right)+\ldots +y\left(i+W_{p}/2\right))$$

and two thresholds are defined as:

(7) $$THR_{1}=MA_{b}\left(i\right).$$

(8) $$THR_{2}=\left|{\bar{W}}_{p}\right|,$$

where $|{\bar{W}}_{p}|$ is the average window size of the peak periods within a certain range. As discussed, the average signal amplitude of peak period is usually higher than that of beat period and the systolic peak is usually the highest value within the peak period. So the systolic peaks can be detected with such conditions:

- $y(i_{1})$, $y(i_{2})$, $y(i_{3})$… are considered as the block of interest if $MA_{p}\left(i_{N}\right)$ is larger than $THR_{1}$
- The block of interest is discarded, if the width of the block is smaller than $THR_{2}$. The $THR_{2}$ is calculated as $(W_{p}\left(i_{1}\right)+(W_{p}\left(i_{2}\right)+(W_{p}\left(i_{3}\right)+\ldots +(W_{p}\left(i_{N}\right))/N$.
- The peaks are the maximum values in the blocks of interest.

Since the BVP signal has been very well filtered by PVM method, the only parameters that have to be determined are the window sizes of the beat periods and peak periods. We applied Fast Fourier Transform (FFT) on the BVP signal over a 10 s’ window and get the frequency $F_{b}\left(i\right)$ for each point in time and then the $W_{b}\left(i\right)$ is calculated as $1/F_{b}\left(i\right)$. For the detrended BVP signal, the peaks usually appear in the positive part that is approximately half of the signal. The peak period should be within the beat period, therefore we can set the window size of the peak period as half of the positive part of the beat period which is 0.25 times of the beat period so the value of $W_{p}\left(i\right)$ is 0.25 ×$W_{b}\left(i\right)$ for each point.

**Figure 1:**
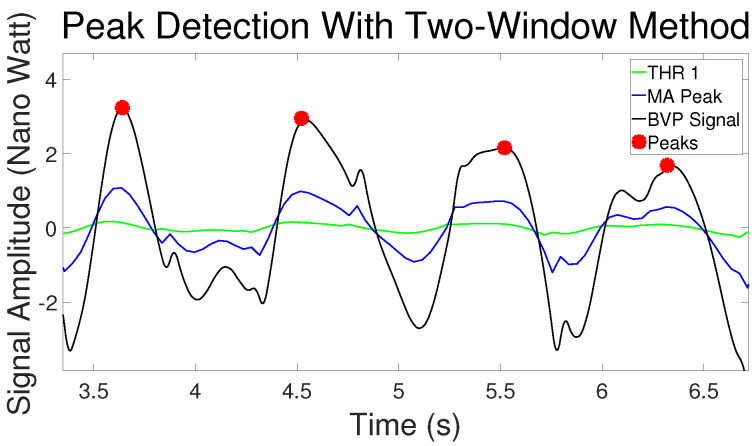
An example of peak detection with Two-Window method.

Figure 1 shows an example of the peak detection of the BVP signal with the Two-Window method. The black curve is the BVP signal generated from the MMSE dataset [24]. The blue curve is the moving average of the systolic peak area ($MA_{p}$). The green curve is the threshold ($THR_{1}$). It can be seen that in this case, the false peaks between the first and second beat periods are eliminated because the $MA_{p}$ is not higher than the $THR_{1}$. So this part is considered noise.

## 4. Experiments

The new algorithm was tested and compared with the state-of-the-art peak detection methods in the framework of remote PRV measurement with MMSE dataset created by Zhang et al. [24].

### 4.1. MMSE Dataset

**Figure 2:**
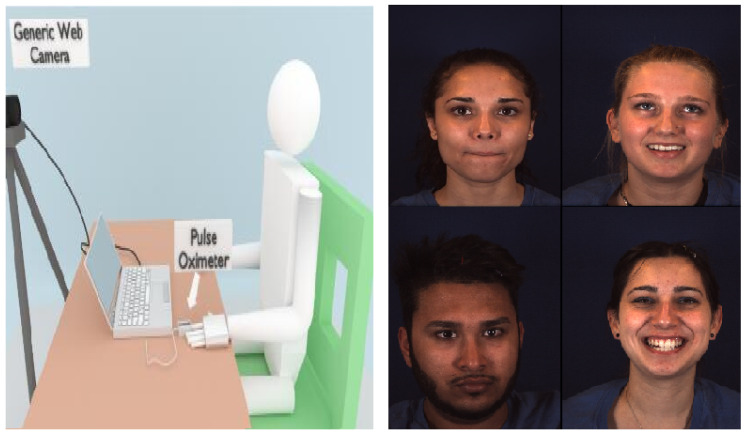
Experimental set up and some sample images from the MMSE database.

For the experiments, we used the MMSE dataset which consists of about 100 videos recorded by RGB cameras with the frame rate of 25 Hz. This dataset was chosen for the following reasons: first of all, the dataset is large enough for training and testing. Secondly, the dataset includes different peoples, such as Europeans, Middle Easterners, South Asians, South Americans and East Asians which potentially makes it more challenging for the RGB signal processing. Thirdly, the contact PPG sensor was used to record the pulse signal as the ground truth which was synchronized with the videos so that it can be used to quantitatively assess the results. Lastly, with emotion elicitation in the experiments, the volunteers showed some movement of faces and heads which make the dataset more complicated and closer to the realistic scenarios. Similar to all the other rPPG experiments, the volunteers were asked to sit at a fixed distance from the web camera with a background board. A simplified description of the experiment set up and several sample images are shown in Figure 2.

### 4.2. The State-of-the-Art Methods for BVP Peak Detection

In the experiments, we assessed two state-of-the-art BVP peak detection methods as reference, namely the Local Maximum method and the SSF method.

#### 4.2.1. Local Maximum

The Local Maximum detection with rules [20] is the most straightforward method for peak detection of BVP signals. Fortunately, the MATLAB function *findpeaks* has provided us with several useful features:

- *MinPeakHeight*: the minimum height of detected peaks.
- *MinPeakDistance*: the minimum distance between detected peaks.
- *MinPeakProminence*: the minimum height of the peaks relative to the lowest bottom line within a certain range.

To detect the peaks of the remotely measured BVP signal with *findpeaks*, we used the brute force search to determine the values of the parameters. The ground truth of peak locations are given by the contact PPG. We used the number of correctly detected peaks with regards to the ground truth, the number of false and missing peaks with regards to the ground truth, and the average errors of peak locations to optimize the parameters. With the the MMSE dataset, 37 videos were used to get the parameters, and the other 54 videos were used to test the methods. As a result, the *MinPeakHeight* was set as $0.75*|\bar{y}|$ and the *MinPeakProminence* was $0.3*|\bar{y}|$, where $|\bar{y}|$ is the average absolute value of the detrended BVP signal. The *MinPeakDistance* was set as 0.24 s.

**Figure 3:**
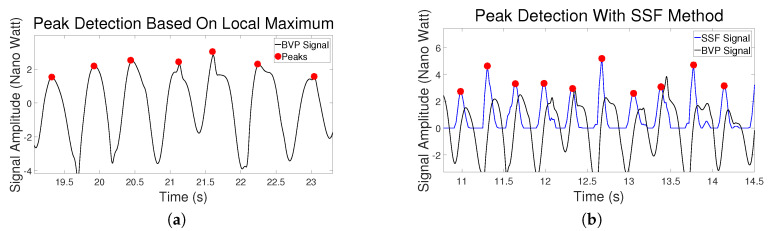
(**a**) An example of Local Maximum method. (**b**) An example of the Slope Sum Function (SSF) method. The original BVP signal is black and the SSF signal is blue.

Figure 3a shows an example of the peak detection based on Local Maximum. It can be seen that it functioned effectively in this case.

#### 4.2.2. Slope Sum Function (SSF)

Zong et al. [22] proposed to use slope sum function (SSF) to detect the onset of Arterial Blood Pressure Pulse by enhancing the rising part of the signal and reducing the descent part. Since remotely measured BVP signals are periodicity exhibiting signals, it is reasonable to adopt this idea to reduce the noise of the signal and make it clearer to detect the peaks [9,23]. The slope sum function is expressed as:

(9) $$SSF\left(i\right)=\sum_{i-w}^{i}\Delta y_{i}\;\;\;\mathrm{and}\;\;\;i=w+1,w+2,\ldots ,N$$

and $\Delta y_{k}$ is expressed as:

(10) $$\Delta y_{i}=\left\{\begin{array}{lll} y(i)-y(i-1) & \mathrm{if} & y(i)-y(i-1)>0\\ 0 & \mathrm{if} & y(i)-y(i-1)\leq 0. \end{array}\right.$$

Equations (9) and (10) show the calculation of the new signal SSF(i) transformed from the original signal, where y is the original signal, *i* represents the time index of the signal and *w* is the window size.

To maximize SSF, the window size *w* should be approximately the same with the length of the rising phase of the original signal. Similar to the Local Maximum method described in Section 4.2.1, this window size and the minimum height were determined by the brute force search. The exact value of *w* was 0.4 s. The threshold height was 0.175 * $|\bar{y}{|}_{ssf}$, where $|\bar{y}{|}_{ssf}$ is the average absolute value of the detrended new signal. The minimum peak distance was 0.24 s.

Figure 3b shows an example of the pre-filtered BVP signal transformed by SSF. The black curve is the BVP signal generated from the MMSE dataset. The blue curve is the new signal after it got processed by SSF. It can be seen that after using the SSF, the upslope part is enhanced and it becomes sharper and more straightforward for peak detection.

### 4.3. System Framework

**Figure 4:**
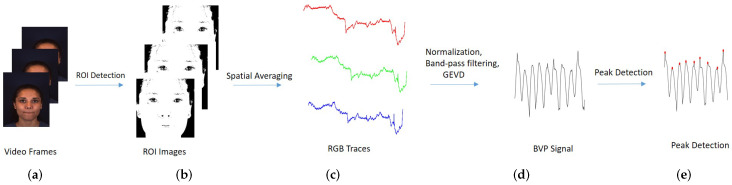
System Framework. (**a**) The original video frames. (**b**) The detected skins. The white part is the detected skin pixels and the black part is the non-skin pixels. (**c**) Spatially averaged RGB signals. (**d**) BVP signal. (**e**) Peak detection.

Our system framework of remote PRV measurement is presented in Figure 4. To get the temporal RGB signals from the video frames, firstly the Viola-Jones face detector and the Kanade-Lucas-Tomasi tracker provided by the OpenCV toolbox were used to get stable facial regions. Then the facial landmarks implemented by Dlib C++ Library [25] were applied to the regions to get the coordinates of corners. The region of interest was cropped based on these corners. Then we used Conaire’s method [26] to detect the skin pixels and discard the non-skin pixels. These skin pixels were spatially averaged to obtain the 3-band RGB signals. The one-dimensional Blood Volume Pulse (BVP) signal was obtained using the newly proposed PVM method as explained in Section 3.1. Then the peak detection methods such as Slope Sum Function (SSF), Adaptive Two-Window method and Local Maximum were used to get the peaks of BVP. With the peaks, the inter-beat intervals (IBI) can be calculated. According to Malik et al. [1], the PRV can be represented in two different ways. It is either calculated as a peak interval series versus number of progressive peaks, or a peak interval series versus time, which is obtained as a signal of a function of time by interpolating the discrete event series (DES). We chose the latter, because in video analysis the FFT is usually utilized to extract the frequency features, and the FFT can only be used with evenly sampled data. In our case, the PRV was obtained by interpolating the IBI signal with the frame rate of 200 Hz.

## 5. Results

### 5.1. Evaluation Metrics

The ground truth of the BVP signal and peak locations were given by the contact sensor, so the precision of the peak detection and PRV measurement can be assessed by the errors between the rPPG signals and the ground truth. The evaluation metrics of this paper are shown in Table 1, which are classified as three groups of metrics:

(1) Peak Detection Errors. This group of metrics is used to evaluate the accuracy of peak detection.

- Peak Location Errors (**PLE(s)**). It is calculated as the average absolute difference between the peak locations detected on the rPPG signal and the annotated peaks of the ground truth.
- Proportion of correctly/incorrectly detected peaks and missing peaks (**%CP**, **%IP** and **%MP**). Since the ground truth is provided by the contact sensor measured from fingers and the rPPG was measured from faces, there is a time difference between the peaks on the rPPG signal and the contact PPG signal which is possibly caused by the different distance from the heart and the recording sensor. As a result, the search range for the correctly detected peaks was set to 0.2 s. If there is more than one peak in the search range, then the extra peaks are considered as incorrectly detected peaks. If there is no peak, then it is considered as a missing peak. With these conditions, **%CP** is calculated as the number of correctly detected peaks over the number of peaks of ground truth. **%IP** and **%MP** are calculated in the same way.

(2) PRV Errors. This group of metrics is used to evaluate the accuracy of PRV measurement.

- PRV Errors (${\mathrm{PRV}}_{\mathrm{er}}\left(s\right)$) and Inter-beat Interval Errors ((${\mathrm{IBI}}_{\mathrm{er}}\left(s\right)$). PRV is obtained as the peak interval series over time interpolated with the frame rate of 200 Hz. IBI is the peak interval series versus number of progressive peaks. Both ${\mathrm{PRV}}_{\mathrm{er}}\left(s\right)$ and ${\mathrm{IBI}}_{\mathrm{er}}\left(s\right)$ are calculated as the absolute average difference between the rPPG signal and gound truth contact PPG signal.
- Relative PRV Errors (${\%\mathrm{PRV}}_{\mathrm{er}}$). It is calculated as the average value of ${\mathrm{PRV}}_{\mathrm{er}}\left(s\right)$ over the PRV of the ground truth.

(3) PRV Feature Errors. This group of metrics is used to evaluate the accuracy of PRV features.

- Errors of Standard Deviation of IBI Series (${\mathrm{STD}}_{\mathrm{er}}\left(s\right)$). This is calculated as the absolute difference between the Standard Deviation (STD) of rPPG measured IBI and the STD of IBI measured by ground truth contact PPG signal.
- Errors of Root Mean Square of Successive Inter-Beat Interval Differences (RMSSD) (${\mathrm{RMSSD}}_{\mathrm{er}}\left(s\right)$). As before, this metric is calculated as the absolute difference between the RMSSD measured by rPPG and the RMMSSD measured by the ground truth. The RMSSD was defined as:
  (11) $$RMSSD=\sqrt{\frac{1}{N-1}\left(\sum_{i=1}^{N-1}{\left(IBI_{\left(i+1\right)}-IBI_{i}\right)}^{2}\right)}$$
  where $IBI_{i}$ is the *i*th peak interval value.

The experimental results were the average values of the entire testing dataset (54 videos).

### 5.2. Results and Discussion

**Figure 5:**
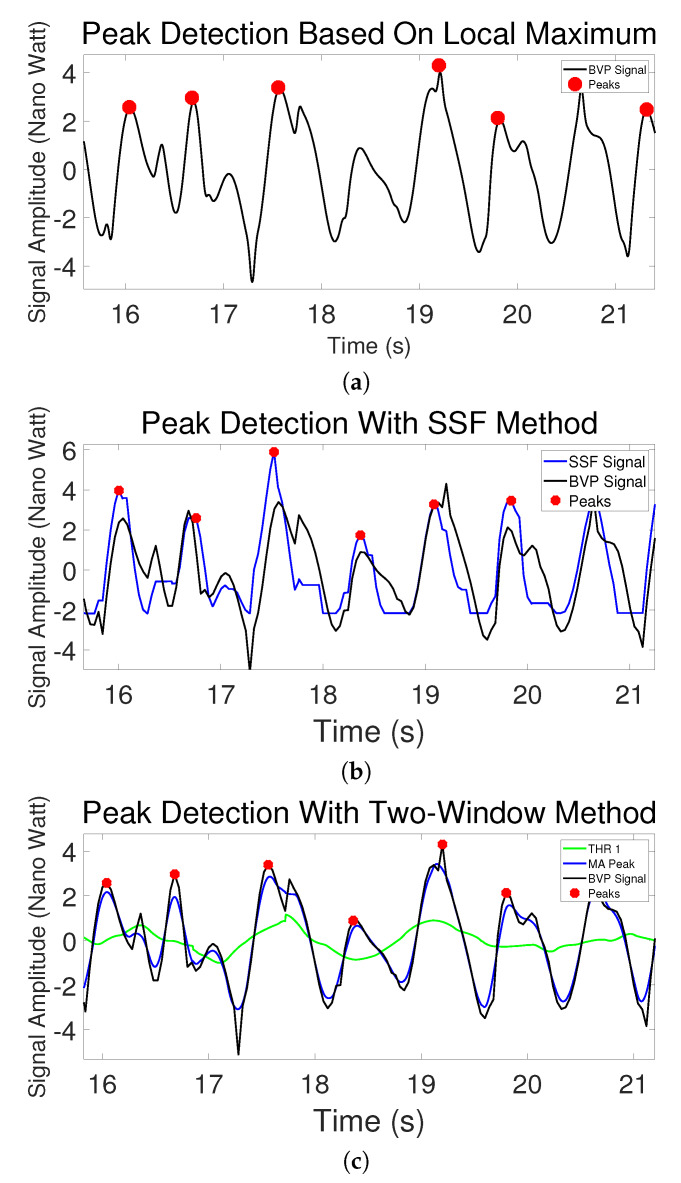
Peak detection on rPPG signal (BVP) with (**a**) Local Maximum, (**b**) SSF and (**c**) Two-Window methods.

**Figure 6:**
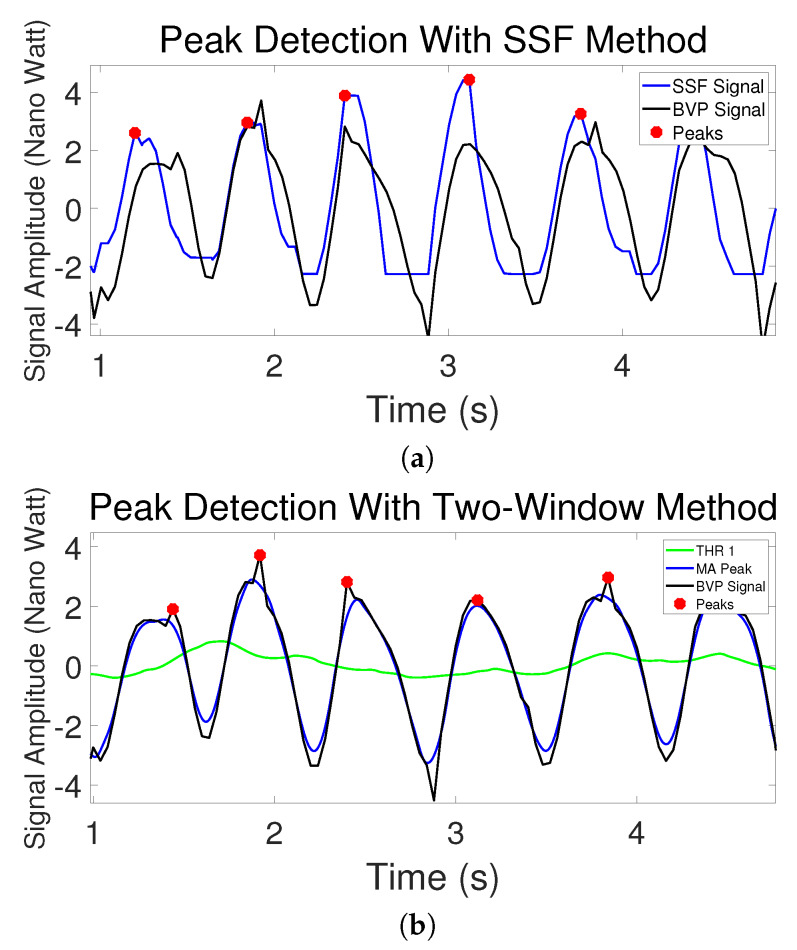
(**a**) SSF in peak detection. (**b**) Two-Window method in peak detection.

Table 2 shows the errors of the peak detection from the three methods. The first column is average peak location errors (**PLE(s)**). It can be seen that the Two-Window method performed better than Local Maximum with much smaller error. The SSF has no result in this column because there is a shift between the new signal transformed by SSF and the original signal due to shape change, as can be seen in Figure 3b, Figure 5b and Figure 6a. Therefore, it is not fair to compare SSF with other methods with location errors. The second, third and fourth columns are the proportion of correctly detected peaks/missing peaks/incorrectly detected peaks. These columns show that the Adaptive Two-Window method gives more peaks correctly detected than the other two methods.

The Local Maximum method worked very well in the majority of the cases after we used the brute force search to determine the parameters, as shown in Figure 3a. However, the results of Table 2 do indicate that the Local Maximum method has a higher probability to fail. Figure 5 shows a typical example where the Local Maximum failed but SSF and Two-Window methods were effective. In this specific case, the Local Maximum did not detect the peak between the 18th second and the 19th second because the height of the beat period is significantly lower than the average height of the entire signal which makes the peak value lower than the parameter “*MinPeakHeight*”. This case shows the weakness of this method. For all such rule-based Local Maximum methods, the physical properties of the BVP curve itself are ignored, and the parameters of the rules are either set manually or using optimization approaches, and it is effective in most of the cases, but it could fail in a few specific cases even if the parameters are fully optimized. On the other hand, the Two-Window method utilizes the physical property that the average height of the systolic peak period is higher than that of the beat period, so a sudden decline of the curve does not affect the performance of the algorithm.

Figure 5b shows why the SSF cannot perform the precise location detection. The SSF is effective because it transforms the signal with an enhanced increasing trend, and thus it avoids losing the fourth systolic peak in this case. However, with the transformation, the shape of the original signal was changed, so the detected locations are slightly more distant from the original peaks. We used this method because the PRV calculation may not be affected if the shift is close to a constant for every peak.

Table 3 shows the results of PRV measurement errors. The IBI is the peak intervals versus number of progressive peaks and the PRV is the peak intervals versus time and is obtained by interpolating the interval series with the time stamps of 200 Hz. According to the table, the Adaptive Two-Window method generated better results for all the three PRV metrics than the other two methods with smaller errors. And the SSF is better than the Local Maximum method. As explained, the PRV measurement is more difficult than the HR measurement and is the key work of this paper. Thus the better results in PRV measurement are significant achievements and prove the advantages of this new algorithm. The table shows that the results of the PRV obtained by the SSF method are worse than the Two-Window method although it performed better than the Local Maximum, and it means the shift caused by SSF transformation is not constant and the location errors are not perfectly reduced in the decrement calculation of the locations.

Figure 6a shows an example where the IBI calculation of SSF is incorrect. It can be seen that the peaks detected between the first and the fourth second on the SSF signal are at the left side of the peaks of the original BVP signal. And the distance between the first peak on the SSF signal and the first peak on the original BVP signal is much larger than the distance between the second pair of the peaks on the two signals. As a result, the IBI calculated by the first peak location and the second peak location with the SSF method is larger than the real value. On the other hand, the peak detection within the same period performed by Adaptive Two-Window method does not have this problem.

The features of the PRV are used in some applications such as emotion recognition. We calculated the errors of the two time domain features (${\mathrm{STD}}_{\mathrm{er}}\left(s\right)$ and ${\mathrm{RMSSD}}_{\mathrm{er}}\left(s\right)$). The results are shown in Table 4 and the 95% Confidence Interval of the PRV features’ values are shown in Table 5. According to the tables, the Adaptive Two-Window performed the best in both metrics with smaller errors. The Confidence Interval of Adaptive Two-Window method is closest to the ground truth compared with the other two methods. And the Local Maximum performed the worst. It should be noted that although our algorithm has significantly improved the remote PRV measurement compared with the other methods, the errors are still large. This is possibly caused by the low frame rate and the noise of the environment, cameras, and so forth. It means the remotely measured PRV cannot replace HRV in critical medical analysis.

**Figure 7:**
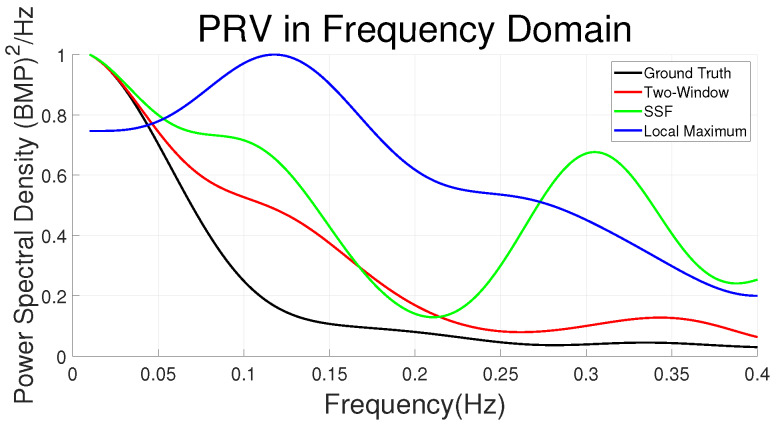
Comparison of PRV measurement with Local Maximum, SSF and Two-Window methods in the frequency domain. The black curve is the ground truth. The red curve is the Two-Window measured signal. The green curve is the SSF measured signal. The blue curve is the Local Maximum measured signal.

The measured PRV in the frequency domain can be evaluated by calculating the Power Spectral Density (PSD) and compared it with the ground truth. Figure 7 shows an example. The black curve is the PSD curve of the ground truth measured by contact PPG. The red/green/blue curves are the PSD signals measured by the Adaptive Two-Window method, SSF method and Local Maximum method respectively. The area within a certain frequency range represents the power of the frequency band. For instance, the area within 0.04 HZ to 0.15 HZ represents the power of the Low Frequency (LF) part which symbolizes the stress state, and the area within 0.15 HZ to 0.4 HZ represents the power of High Frequency (HF) part which symbolizes the relaxed state. Thus the ideal rPPG measured PRV signal should match perfectly with the ground truth signal in the frequency domain. The duration of the videos of the MMSE dataset is very short and some of them are only 20 s so it would be difficult to evaluate the LF part quantitatively. But the results can still be assessed visually. According to the figure, the Adaptive Two-Window method performed better than the other two methods in this case. The SSF method is worse than the Two-Window method with bigger errors in both LF part and HF part and the Local Maximum method performed the worst with very large error in LF part.

## 6. Conclusions and Future Work

Biomedical researchers have shown that the Heart Rate Variability (HRV) and Pulse Rate Variability (PRV) are vital physiological signals for many applications such as stress detection, emotion recognition and recovery monitoring. Computer Vision researchers have shown that the Heart Rate (HR) can be remotely measured based on remote photoplethysmography (rPPG) technique with very high accuracy. However, the precision of remote PRV measurement has not been studied intensively and needs further improvement. In this paper, we proposed to use the Periodic Variance Maximization (PVM) method to extract the remotely measured Blood Volume Pulse (BVP) signal and the Adaptive Two-Window Peak Detection to improve the precision of the peak detection of BVP so that we improved the remote PRV measurement. We tested the algorithm with the MMSE dataset which is recorded with the low-cost RGB camera and quantitatively compared this method with the Local Maximum method and Slope Sum Function method with ten metrics. The results showed that the Adaptive Two-Window method performed better than the other two methods so that our new algorithm can be potentially used in the realistic PRV applications.

However, the results also showed that the relative precision of the PRV measurement is not higher than 90%. A possible approach to address the issues in the future is to study the physical properties of the noise, combine all the methods and propose detailed global rules for the BVP filtering and peak detection for different dataset so that the achievements can be used in realistic applications such as long-term health monitoring, lie detection, and so forth. Another issue is that the PRV measured by rPPG cannot yet replace HRV in critical medical applications, therefore it is important to produce a dataset with the ground truth provided by ECG and improve the remotely measured PRV based on this ground truth.

## Figures and Tables

**Table 1:** Evaluation Metrics.

Category	Metrics	Denotation	Unit
Peak Detection Errors	Peak Location Errors Proportion of correctly detected peaks Proportion of incorrectly detected peaks Proportion of missing peaks	**PLE(s)** **%CP** **%IP** **%MP**	Seconds (s) Percentage (%) Percentage (%) Percentage (%)
PRV Errors	Inter-beat interval Errors PRV Errors Relative PRV Errors	${\mathrm{IBI}}_{\mathrm{er}}\left(s\right)$ ${\mathrm{PRV}}_{\mathrm{er}}\left(s\right)$ ${\%\mathrm{PRV}}_{\mathrm{er}}$	Seconds (s) Seconds (s) Percentage (%)
PRV Feature Errors	Errors of Standard Deviation of IBI signal Errors of Root Mean Square of Successive Inter-Beat Interval Differences (RMSSD)	${\mathrm{STD}}_{\mathrm{er}}\left(s\right)$ ${\mathrm{RMSSD}}_{\mathrm{er}}\left(s\right)$	Seconds (s) Seconds (s)

**Table 2:** The average peak detection errors.

Methods	PLE(s)	%CP	%MP	%IP
*Local Maximum*	0.1423	87.84%	3.770%	8.390%
*SSF*	X	90.53%	4.030%	6.310%
*Two-Window*	0.1221	94.02%	1.960%	4.020%

**Table 3:** The average PRV errors.

Methods	${\mathrm{IBI}}_{\mathrm{er}}$(s)	${\mathrm{PRV}}_{\mathrm{er}}$(s)	%${\mathrm{PRV}}_{\mathrm{er}}$
*Local Maximum*	0.1718	0.1574	21.74%
*SSF*	0.1510	0.1413	21.56%
*Two-Window*	0.1407	0.1185	17.03%

**Table 4:** The average errors of Pulse Rate Variability (PRV) features.

Methods	${\mathrm{STD}}_{\mathrm{er}}$(s)	${\mathrm{RMSSD}}_{\mathrm{er}}$(s)
*Local Maximum*	0.0938	0.1072
*SSF*	0.0781	0.0718
*Two-Window*	0.0511	0.0664

**Table 5:** The 95% Confidence Interval of the PRV features’ values.

Methods	STD (95%ConfidenceInterval) (s)	RMMSD (95%ConfidenceInterval) (s)
*Ground Truth*	0.0631±0.0067	0.0670±0.0097
*Local Maximum*	0.1570±0.0140	0.1742±0.0173
*SSF*	0.1412±0.0181	0.1388±0.0229
*Two-Window*	0.1142±0.0133	0.1334±0.0185
